# Molecular Phylogeny of Tribe Theeae (Theaceae *s.s*.) and Its Implications for Generic Delimitation

**DOI:** 10.1371/journal.pone.0098133

**Published:** 2014-05-21

**Authors:** Wei Zhang, Sheng-long Kan, Hong Zhao, Zhen-yu Li, Xiao-quan Wang

**Affiliations:** 1 Marine College, Shandong University at Weihai, Weihai, China; 2 State Key Laboratory of Systematic and Evolutionary Botany, Institute of Botany, Chinese Academy of Sciences, Beijing, China; Royal Botanic Gardens, Kew, United Kingdom

## Abstract

Tribe Theeae, which includes some economically important and widely grown plants, such as beverage tea and a number of woody ornamentals, is the largest member of the Theaceae family. Using five genomic regions (chloroplast: *atpI-H*, *matK*, *psbA5'R-ALS-11F*, *rbcL*; nuclear: *LEAFY*) and 30 species representing four of the five genera in this tribe (*Apterosperma*, *Camellia*, *Polyspora*, and *Pyrenaria* s.l.), we investigated the phylogeny of Theeae and assessed the delimitation of genera in the tribe. Our results showed that *Polyspora* was monophyletic and the sister of the three other genera of Theeae investigated, *Camellia* was paraphyletic and *Pyrenaria* was polyphyletic. The inconsistent phylogenetic placement of some species of Theeae between the nuclear and chloroplast trees suggested widespread hybridization between *Camellia* and *Pyrenaria*, *Polyspora* and *Parapyrenaria*. These results indicate that hybridization, rather than morphological homoplasy, has confused the current classification of Theeae. In addition, the phylogenetic placement and possible allies of *Laplacea* are also discussed.

## Introduction

Tribe Theeae Szyszylowicz, comprising more than 400 species and with a variety of morphological diversity [1], [2], is the largest group in the family Theaceae Ker Gawl. It is mainly distributed in eastern and southeastern Asia, with southwest China as the center of species diversity [1]. Species of this tribe are often economically important and the most well known species are beverage tea (*Camellia sinensis* (L.) Kuntze), the cooking oil tree (*C. oleifera* Abel) and a number of woody ornamentals (e.g. *C. japonica* L., *Polyspora axillaris* Sweet and *Apterosperma oblata* Chang). Recently, there has been a growing interest in the medicinal and health benefits of members in this group, especially species of *C. sinensis*, which have shown real potential in the treatment of cancer, diabetes, obesity and many cardiovascular ailments. It is therefore critical to understand the phylogenetic relationships among these species in order to advance breeding strategies, conservation strategies and the discovery of potentially medicinal plants. Despite extensive morphological and anatomical investigations, however, the taxonomy within the Theeae remains confused [3], [4].

Theeae historically included many more taxa than currently circumscribed. De Candolle (1824) first published Camellieae ( = Theeae) to accommodate only two genera: *Camellia* L. and *Thea* L. (considered synonymous by later authors) [5]. As more and more species were discovered, the number of genera correspondingly increased. In *Genera Plantarum*, Bentham and Hooker (1862) expanded the scope of the family Theaceae to include six tribes and 32 genera, but transferred the genus *Camellia* from tribe Camellieae DC. to Gordonieae DC. [6]. Szyszylowicz (1895) excluded a great number of tribes and genera from Theaceae and limited the family to only 16 genera. He also established the new tribe Theeae based on the following genera: *Gordonia* Ellis, *Haemocharis* Salisb. ( = *Laplacea* Kunth), *Pyrenaria* Blume, *Schima* Reinw. ex Blume, *Stewartia* L. and *Thea* L. ( = *Camellia* L.) [7]. This taxonomic treatment was debatable, however. Some authors, such as Melchior, Airy-Shaw, Sealy, Keng and Tsou, often used Camellieae [8]–[11], while other authors, such as Ye, Takhtajan and Chang, preferred Theeae [12]–[14]. Using molecular data, Prince & Parks and Yang et al. confirmed that the currently defined Theeae forms a clade [15], [16]. However, the generic delimitations and the phylogenetic relationships among the genera within Theeae, especially the genera from Asia, remain confused and unresolved. *Camellia s.l*. has been split into numbers of genera since it was first established by Linnaeus (1753), while *Pyrenaria* now includes *Tutcheria* Dunn, *Sinopyrenaria* Hu and *Parapyrenaria* Chang [17], [18]. *Polyspora* Sweet has been separated from *Gordonia* in order to accommodate the Asian species [12], [15], [16], Whereas *Apterosperma* Chang still has an uncertain systematic position, often being placed into tribe Schimeae Ye ( = Gordonieae Tsou) or Theeae [15], [16], [19].

The paucity of morphological diagnostic characters, together with their overlapping and convergent nature were once blamed for the taxonomic confusion [11]. Although the number of bracteoles and degree of differentiation between sepals and petals were used as key characters among these genera [8], [9], they vary considerably within and continuously between genera. For example, the flowers of *C. sinensis*, have 2 or 3 bracteoles, 5 or 6 sepals abruptly distinct from the 7 or 8 petals and the parts nearly to fully distinct, differ from those of *C. wenshanensis* Hu, which have 10 perules ( = bracteoles+sepals, with a gradual progression in size and shape) that are incompletely distinct from the petals. Individually, it has been suggested that the seed and fruit traits are stable among these genera but conflict with each other when considered together. For example, *Camellia* and *Pyrenaria* have similar seed forms (without wings), while *Camellia* and *Polyspora* have similar dehiscent fruits. Additional embryological evidence has supported the close relationship between *Camellia*, *Polyspora* and *Pyrenaria* but has excluded *Apterosperma* from Theeae [11]. Overall, the morphological data have not successfully addressed the relationships and taxonomic boundaries within Theeae. Perhaps its classification is not only a problem of methodology (morphological characters selected, or the way they are analyzed), but also an intrinsic feature of the tribe resulting from its particular evolution.

Current molecular phylogenetic studies recognize the monophyly of Theeae, which includes *Apterosperma*, *Camellia*, *Laplacea*, *Polyspora* and *Pyrenaria*, but leaves relationships between them unresolved and controversial [15]–[17], [20], [21]. For example, Prince & Parks, using chloroplast coding genes (*matK* and *rbcL*), recognized *Polyspora* as sister to the remaining taxa within Theeae [15]. This is in contrast to the nuclear DNA data (ITS), which showed that *Camellia* and *Polyspora* to be closer to each other than they are to *Pyrenaria* (*Tutcheria*) [16], [17]. *Camellia* is the key genus among these taxonomic problems. Phylogenetic studies of *Camellia* using sequences from the chloroplast genome [15], [22], mitochondrial genome [23] and nuclear sequence data [16], [17] were unsuccessfully in uniting the representatives of *Camellia* into a monophyletic lineage, raising doubts about the monophyly of *Camellia*.

This study presents a molecular phylogeny of tribe Theeae, based on sequences from the chloroplast markers *atpI-H*, *matK*, *psbA5'R-ALS-11F* and *rbcL*, and the nuclear gene *LEAFY* in order to: (1) reassess the generic boundaries and phylogenetic relationships within Theeae and (2) explore the reasons for the previous confusing classification within Theeae.

## Materials and Methods

### Taxon sampling

We followed the tribal description used by Prince & Parks [15] and recognized *Apterosperma*, *Camellia*, *Laplacea*, *Polyspora* and *Pyrenaria s.l*. as being members of this tribe. *Parapyrenaria*, *Pyrenaria s.s*. and *Tutcheria* have recently been reduced into *Pyrenaria s.l*. [1]. However, we provisionally retained them herein to make comparisons with previous investigations. We sampled a total of 30 taxa: *Camellia* (18 species), *Polyspora s.l*. (three species), *Tutcheria* (seven species) and two monotypic genera *Apterosperma* and *Parapyrenaria*. In addition, *matK* and *rbcL* sequences for 11 additional species, including five *Camellia*, two *Laplacea* and four *Polyspora s.l.* were obtained from GenBank (Table S1). Polyploids are relatively common in Theeae, particularly in the species-rich genus *Camellia*, among which 34% of the species have been reported to be polyploids [24]–[26]. Under natural conditions, the ploidy of *Camellia* included tetraploids, hexaploids and octoploids. Furthermore, a series of ploidy numbers was found in different populations within the same species. For example, *C. forrestii* (Diels) Coh. St. consists of diploids, tetraploids and hexaploids in different populations [26]. To avoid the possibility of phylogenetic incongruence resulting from the polyploid species, we decided to select only diploid species that have been confirmed by previous cytological studies [26]–[31]. In addition, species were selected, as far as possible, to maximize the coverage of morphological diversity. For example, based on the recent classification of *Camellia*, the 18 species of *Camellia* selected covered both subgenera and six of the 14 sections of this genus [1]. *Laplacea* material are difficult to sample in the wild, and although the DNA from herbarium specimens (voucher numbers are Hatschbach 48333 and Sun 749 in PE herbarium) were extracted they were failed with PCRs. Thanks to the sequences deposited in GenBank, two species of *Laplacea* were included in this study, and their phylogenetic positions were analyzed using an additional *mark*+*rbcL* matrice. *Stewartia* as the potential outgroup was determined from previous phylogenetic studies [15], [16].

### DNA extraction, amplification, cloning and sequencing

Total DNA was isolated from silica gel-dried leaves, following the modified CTAB method [32], and was used as a template in the polymerase chain reaction (PCR). Primers for amplification and sequencing of the *atpI-H* and *psbA5'R-ALS-11F* chloroplast regions followed Shaw et al. [33], [34]. Another two primers for the *matK* and *rbcL* regions were modified from a previous publication so that they were specific to Theeae [15] (Table S2). The second intron of the *LEAFY* gene was originally amplified and sequenced using degenerate LFsxl-2 and LFtxr primers [35], and the designs of the Theeae specific primers were based on these sequences (Table S2). The PCR amplification products from the chloroplast regions were purified using a TIANgel Midi Purification Kit (Tiangen Biotech, Beijing Co., LTD) and sequenced on a 96-capillary 3730XL DNA analyzer (Applied Biosystems, Foster City, CA, USA). The amplified fragments of the *LEAFY* gene were electrophoresed in 1.5% agarose gel and purified using a TIANgel Midi Purification Kit (Tiangen Biotech, Beijing Co., LTD). Direct sequencing identified homozygotes and heterozygotes. Homozygous sequences were directly incorporated into the alignment, whereas heterozygous sequences were cloned using the pGEM-T EASY Vector System II (Progmega). Six to eight clones per individuals were selected and bi-directionally sequenced with the primer T7 and SP6.

### Phylogenetic analyses

Sequence alignments were initially performed with ClustalX [36] and adjusted manually using BioEdit 7.0.5 [37]. The four cpDNA regions were analyzed both separately and in combination in order to assess the congruence between the different cpDNA data matrices. The incongruence length difference test (ILD) [38] was also performed to examine the extent of conflict among different cpDNA data subsets and between chloroplast and nuclear regions. This test was carried out by PAUP4.0b10 [39] in a pairwise fashion, using 1000 replicates with 10 random addition sequences per replicate.

Maximum parsimony (MP), maximum likelihood (ML) and Bayesian inference (BI) methods were used for the phylogenetic analyses. The best fitting models for the sequence evolution of the BI and ML analyses were determined using MrModeltest 2.2 [40] and jModeltest 0.1.1 [41], respectively. MP analysis was conducted using PAUP4.0b10. A heuristic search was performed with 1000 random addition replicates, tree bisection-reconnection (TBR) swapping and the multrees option in the analysis program. Bootstrap analyses, based on 1000 replicates with 10 random additions per replicate were used to estimate the confidence of the clades. Unambiguous indels were treated as phylogenetic characters according to the simple indel coding method [42]. This process was performed by GapCoder [43], and the Gap matrices were only used in MP analyses. ML analysis was conducted using PhyML 3.0 [44] and the online South of France bioinformatics platform (http://www.atgc-montpellier.fr/phyml/). They were performed with the GTR substitution model, estimated gamma shape parameter and BioNJ starting tree implemented options. Bootstraps analysis was performed with 100 replicates using SPR & NNI tree topology search operation. BI inference was undertaken using MrBayes 3.1.2 [45]. In this analysis, the Markov chain Monte Carlo (MCMC) algorithm was run for 1,000,000 generations, with one cold chain and three heated chains, starting from random trees and retained one out of every 100 generations. The first 3000 trees were discarded as a conservation burn-in, and the remaining trees were used to construct the 50% majority rule consensus tree. There were some phylogenetic incongruences among the MP, ML and BI analyses of *matK*+*rbcL* matrice, and thus an additional Neighbor-joining (NJ) analysis was performed by PAUP4.0b10 with 1000 replicates to determine the optimal trees.

### Molecular dating

To infer the age of the major evolutionary lineages, a molecular clock analysis was performed following the practical guide of Sauquet [46]. Prior to the analysis, a likelihood ratio test was carried out to examine the evolutionary rate constancy among lineages [47]. Since the data rejected the assumption of equal rates in sister groups (*P*<0.05), a relaxed Bayesian molecular clock analysis [48] was selected using BEAST 1.4.6 [49]. We used the GTR model of nucleotide substitution with a gamma distribution and four rate categories, under an uncorrelated lognormal relaxed clock model. A yule tree prior was performed as suggested for species level phylogeny [49]. Two independent MCMC analyses of 10,000,000 steps were specified, sampling every 1000 generations, with a burn-in of 1000 (10%) trees, and the results were analyzed using Tracer 1.4.1 [49]. The maximum clade credibility tree was summarized in TreeAnnotator 1.4.8 with a posterior probability limit of 0.5 and node heights for the mean. Finally, the summary tree was viewed and edited in FigTree 1.2.2 [49].

The confusing identification of fossils in this family is not surprising since the classification of family Theaceae is ambiguous. Although many fossils have been reported in the family Theaceae, two fossil records available to us are considered reliable and were used for calibration. One fossil is *Andrewsiocarpon henryense* (IU158154592), an extinct species from the Middle Eocene Claiborne Formation of western Kentucky and Tennessee [50]. *Andrewsiocarpon henryense* had been originally placed in the Gordonieae [4], but in view of its compressed globose capsules and wingless seeds [51], it is now considered closely related to members of tribe Theeae. Therefore, the stem node of Theeae was constrained to a minimum age of 40 mya (using a lognormal prior distribution with a standard deviation of 1.0). The other fossil is *Camellia japonoxyla* (KUN 0027), a species from the Lower Miocene Yanagida Formation of the Noto Peninsula, Japan [52]. Since previous studies have confirmed the systematic placement of this fossil and its age [53]–[55], the stem node of *Camellia* was constrained to a minimum age of 20 mya, with a standard deviation of 1.0.

## Results

### Alignment and sequence characteristics

The *atpI-atpH* fragments, varying in length between 664 and 1074 bp, were the most variable partition among the four cpDNA regions. In contrast, the *psbA5'R-ALS-11F* region, with a 9 bp variation in length, was the least variable partition. Among the four cpDNA regions, *atpI-atpH* provided the highest percentage of both variable characters (8.06%) and parsimony informative characters (PIC, 3.61%), but had the lowest consistency index (CI, 0.7301) and the lowest retention index (RI, 0.7532). The *rbcL* alignment had the lowest percentage number of variable characters (2.78%) and the lowest PIC (1.44%) (Table 1). The length of each four cpDNA regions in the alignment ranged from 679 bp (*psbA5'R-ALS-11F*) to 1458 bp (*matK*). We combined all the cpDNA partitions because of the fact that there was no significant incongruence among the markers, according to the ILD test (*P* = 1). The aligned matrice was 4242 bp in length after 100 bp alignment ambiguities were excluded. Coding the gaps yielded 24 additional characters, producing a total of 4266 characters, including 254 bp (5.95%) variable characters and 93 (2.18%) PICs (Table 1).

**Table 1:** Statistics from the analysis datasets.

Statistic	*atpI-atpH*	*matK*	*psbA5'R-ALS-11F*	*rbcL*	Combined plastid regions	*LEAFY*
length variation (bp)	664–1074	1431–1464	681–690	1028–1041	3811–4257	1215–1418
No. of excluded positions	52	20	23	5	100	453
Aligned length (bp)	1064	1458	679	1041	4242	1554
Indel character	16	4	1	3	24	62
No. of variable characters (%)	87 (8.06)	93 (6.36)	45 (6.62)	29 (2.78)	254 (5.95)	495 (30.63)
No. of parsimony informative characters (%)	39 (3.61)	29 (1.98)	10 (1.47)	15 (1.44)	93 (2.18)	365 (22.59)
No. of shortest trees	2868	3	16	444	476	30
Tree length	128	101	51	36	356	713
CI	0.7031	0.9505	0.9608	0.8333	0.7444	0.7784
RI	0.7532	0.9630	0.9600	0.9032	0.7731	0.9035
Evolutionary model selected	GTR+I+G	GTR+G	GTR+G	F81+G	GTR+I+G	GTR+G
-lnL	2094.0750	2591.6982	1226.9083	1655.5642	7911.7783	6024.7490

The PCR production of the *LEAFY* sequences yielded a single band, representing one or two distinct clones for each individual within a species. All of these clones were sequenced and analyzed. We originally obtained the complete intron 2 sequence for *LEAFY*, but after the universal primers were redesigned and unambiguous end regions were eliminated, the remaining sequences were found to be partial sequences of exon 2 (13 bp) and partial intron 2 sequences. The final fragment of *LEAFY* ranged from 1215 bp to 1418 bp in length. This yielded a total of 1616 characters, as 453 positions were removed as alignment ambiguities and 62 indel-coding characters were added. Compared with the combined cpDNA regions, this partition of the *LEAFY* gene was most informative and provided five times more variable characters (30.63%) and over ten times more PICs (22.59%) (Table 1).

### Phylogenetic analyses

**Figure 1:**
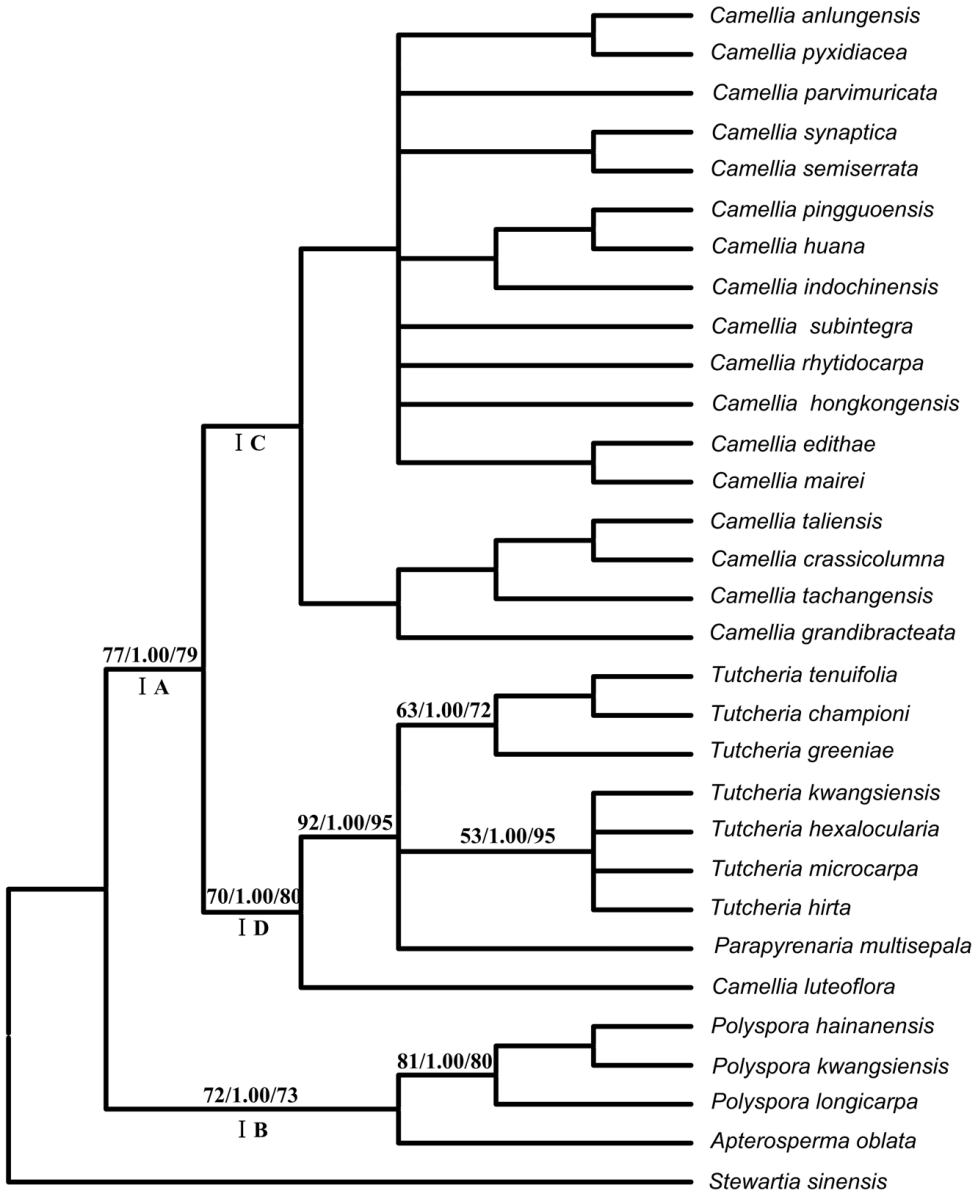
The maximum parsimony tree for the Theeae inferred from the combined cpDNA sequences. Numbers on the branches indicate the bootstrap values for MP (>50%), the Bayesian posterior probabilities (>95%) and the ML bootstrap values (>50%), respectively.

**Figure 2:**
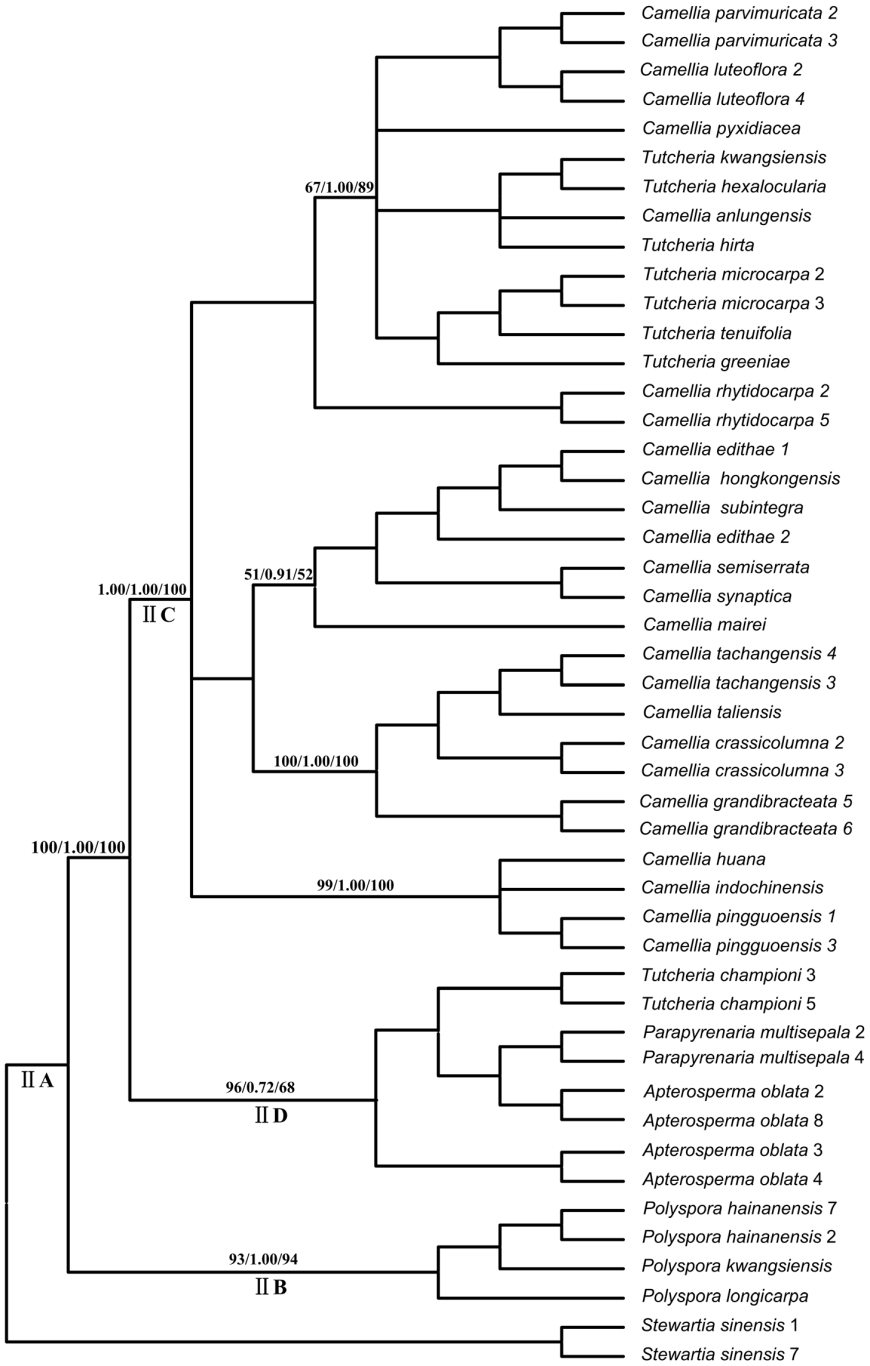
The maximum parsimony tree of the Theeae inferred from the *LEAFY* sequences. Numbers on the branches indicate the bootstrap values for MP (>50%), the Bayesian posterior probabilities (>95%) and the ML bootstrap values (>50%), respectively. Numbers following a species name represent clone numbers.

Both the results of the ILD test (*P* = 0.002) and a comparison of the individual topologies revealed significant incongruences between the cpDNA and the *LEAFY* phylogenies, so the two datasets were analyzed separately. The MP analysis of the combined cpDNA data resulted in 476 shortest trees, each with a length of 356 steps, a CI of 0.7444 and a RI of 0.7731. The MP analysis of the *LEAFY* data yielded 30 minimal length trees, containing 713 steps, with a CI of 0.7444 and a RI of 0.7731. The GTR+I+G and GTR+R models were selected as the best-fit evolutionary models for the combined cpDNA and *LEAFY* data, respectively, which were then used in the subsequent ML, BI and BEAST analyses. Among the tree results, the MP strict consensus tree was identical to the BI and ML topologies (the BI and ML trees are not presented). Only clades with significant support values (bootstrap value >70%; posterior probabilities >0.9) are discussed. In the cpDNA tree, the Theeae were divided into two main clades with moderate support: clade IA (MP/BI/ML:77/1.00/79) and clade IB (72/1.00/73). Clade IB consisted of the two sister genera *Polyspora* and *Apterosperma*. Clade IA comprised two subclades IC and ID. ID included *Tutcheria* and *Parapyrenaria*, with *Camellia luteoflora* as the earliest-diverging taxon. The remaining species of *Camellia* formed the IC clade, but had weak support (<50% BS) (Figure 1). In contrast, the strict consensus tree constructed using *LEAFY* yielded three well-supported clades: IIB, IIC and IID. IIB was made up of *Polyspora* species and was the sister of IIC and IID. IID included *Tutcheria*, *Parapyrenaria* and *Apterosperma*, while IIC mainly comprised species of *Camellia*, with some members of *Tutcheria* was nested within it (Figure 2).

### Incongruence between the cpDNA and the *LEAFY* datasets

The results of the ILD test showed significantly conflicting phylogenetic estimations between cpDNA and *LEAFY* datasets (*P* = 0.002). One of the key contradictory results was the uncertain position of the monotypic genus *Apterosperma*, which was either closest to *Polyspora* or to *Pyrenaria s.l*. (including *Parapyrenaria* and *Tutcheria*) in the cpDNA tree and *LEAFY* tree, respectively (Figure 1 vs. Figure 2). Another key conflicting result was the ambiguous relationships among species of *Pyrenaria s.l*. In the cpDNA tree the *Pyrenaria* species formed a well-supported monophyletic clade (92/1.00/95) (Figure 1). However, in the *LEAFY* tree, some species of *Pyrenaria s.l*. (*Tutcheria*) were nested within *Camellia*, which indicated a polyphyletic *Pyrenaria* (Figure 2). Other differences existed, but these occurred among the clades with low or unresolved phylogenetic resolution, and thus have not been discussed in this paper.

### Molecular dating

**Figure 3:**
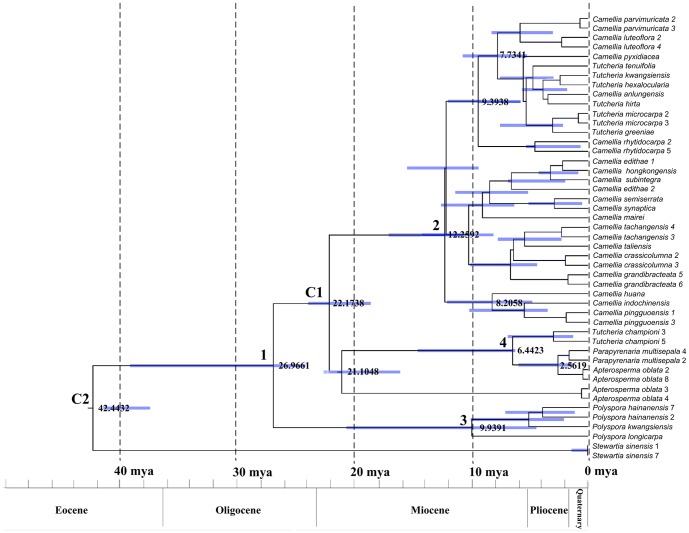
Fossil-calibrated molecular chronogram of Theeae based on the *LEAFY* sequences. Dark gray bars represent 95% confident intervals for nodal ages. Numbers adjacent to the nodes indicate the ages of the nodes of interest (see Table 2). Numbers following a species name represent clone numbers.

The Effective Sample Size (ESS) value for the cpDNA data and *LEAFY* data statistics was 1166.84 and 718.89, respectively, which indicated that there were only a small number of correlated samples and thus may represent the posterior distribution. The molecular dating results derived from the independent data matrices (cpDNA vs. *LEAFY*) were largely consistent with each other (Table 2, Figure 3 vs. Figure S1). Therefore, the MCC chronogram inferred by the *LEAFY* dataset is the only one shown in this paper (Figure 3). The *LEAFY* dating results suggest that the crown group of the *Parapyrenaria* + *Tutcheria* was dated 6.44 mya (95% HPD: 6.23–14.58 mya) and a similar time span was also obtained from the cpDNA dating results (7.27 mya, 95% HPD: 4.51–12.35 mya). The crown age for Theeae was estimated to be 27.00 mya, according to the combined *LEAFY* and cpDNA dating results, which were 26.97 mya (95% HPD: 24.23–39.25 mya) and 27.07 mya (95% HPD: 18.36–34.12 mya), respectively (Table 2, Figure 3). The *Apterosperma* clades, earliest-diverging at 21.10 mya (95% HPD: 14.91–21.48 mya) in the *LEAFY* chronogram and 13.45 mya (95% HPD: 7.22–24.08 mya) in the cpDNA chronogram, showed some dating discrepancies.

**Table 2:** Divergence time estimation of the major Theeae lineages based on BEAST.

Node in Fig.3	Description of node	*LEAFY* gene data	cpDNA data
		(mya)	(mya)
1	Crown of Theeae	26.97 (24.23–39.25)	27.07(18.36–34.12)
2	Crown of *Camellia*	12.26 (10.84–17.08)	15.89 (7.31–18.67)
3	Crown of *Polyspora*	9.94 (4.40–20.71)	6.40 (3.38–15.27)
4	Split between *Parapyrenaria* and *Tutcheria*	6.44 (6.23–14.58)	7.27 (4.51–12.35)
C1	Stem of *Camellia* (Calibration)	22.17 (18.61–23.98)	22.37 (18.00–23.89)
C2	Stem of tribe Theeae (Calibration)	42.44 (37.54–41.54)	40.88 (37.89–43.83)

(numbers given in brackets represent 95% confidence intervals).

## Discussion

### The phylogenetic position of *Laplacea*

**Figure 4:**
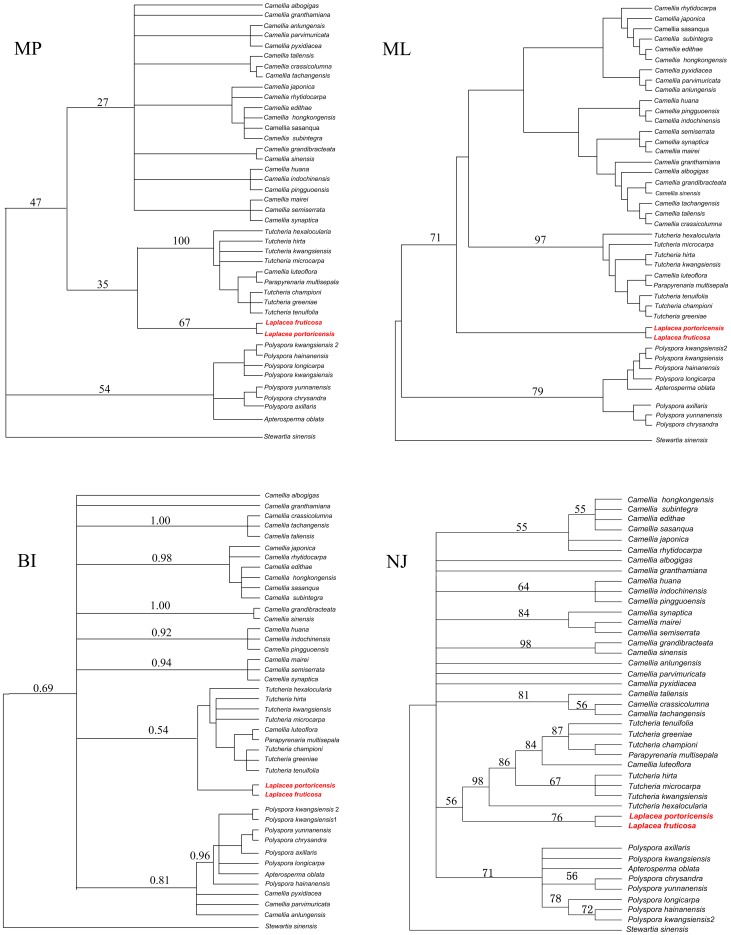
Phylogenetic positions of *Laplacea* from different phylogenetic analyses of *matk*+*rbcL* data sets. Numbers on the branches indicate the bootstrap values for MP (Maximum Parsimony), ML (Maximum Likelihood), NJ (Neighbor Joining) and posterior probabilities for BI (Bayesian Inference).

The New World genus *Laplacea* has been variously described as consisting of from 2 to 20 species [2]. Previous molecular data has placed *Laplacea* within the tribe Theeae, however, left its phylogenetic position unresolved for a long time [15]. Although materials of this genus were unavailable in this study, we constructed phylogenies included all representatives of genera of Theeae using the combined sequence data from our study and GenBank. The results of different tree-building methods showed that the *Laplacea* sisters to the *Camellia* + *Pyrenaria* clade (Figure 4, ML tree) or within this clade and sisters to *Pyrenaria* (Figure 4, MP, BI and NJ trees). Although the weak support values in these results prevented us to draw a firm conclusion, the congruent tree topologies do not supported the taxonomic treatment of Sealy (1958), Keng (1980), Ye (1990), or Takhtajan (1997) since they all considered *Polyspora* and *Laplacea* as more closely related to each other than to other genera in Theeae [10], [13], [56], [57]. Over all, these results improved our understanding of the systematic position and possible allies of *Laplacea*, however, more rigorous evaluations are still necessary in the future.

### Incongruence between the cpDNA and *LEAFY* datasets

The ILD test on the cpDNA vs. *LEAFY* datasets (*P* = 0.002), as well as a visual comparison of their topologies and branch support values (Figure 1 vs. Figure 2), indicated some phylogenetic inconsistencies. Several reasons may account for the phylogenetic incongruence, including technical causes and evolutionary processes [58]. Since *LEAFY* is a well known single-copy gene in angiosperms [59], [60], it is unlikely that ortholog/paralog confusion would give rise to phylogenetic incongruence. Phylogenetic incongruence may also result from long branch attraction, the erroneous grouping of two or more long branches as sister groups due to relatively sparse taxon sampling [61]. However, our MP, ML and BI trees were topologically identical and had no comparatively long branches. Therefore, long branch attraction can be rejected as a plausible explanation. Hybridization and incomplete lineage sorting (ILS) are important biological explanations for phylogenetic incongruence and are often difficult to distinguish from each other [62], [63]. Although there are several statistical approaches for distinguishing hybridization and ILS, the lack of sufficient unlinked genomic data sets and the fact that very little is known about effective population sizes and the generation times of the taxa, limited their use in our study [64], [65]. Instead, we followed Sang & Zhong's assumption that the ILS results from the ancestral alleles being randomly sorted into some lineages [66]. The divergence time for these alleles was prior to the species to which they belong, but different from each other. In contrast, if the incongruence was caused by hybridization, the divergence time for each gene would be identical to those of the species tree. Therefore, the divergence time for each gene between the parental taxa is nearly equal in the case of hybridization, but differs significantly in the case of lineage sorting [66]. Thus, the ILS hypothesis seems less plausible, given the similar molecular dating results that were derived independently from the cpDNA and *LEAFY* datasets. We can therefore infer that hybridization is probably the most plausible explanation for these inconsistencies. However, further analyses with more nuclear markers and more individuals should be conducted to clarify this issue.

### Paraphyly of *Camellia s*.*l*

In this study we assessed the generic delimitation of *Camellia* using chloroplast gene and nuclear gene sequence data. The results confirmed previous analyses showing that *Camellia* is not monophyletic, but includes species of *Pyrenaria s.l*. (including *Parapyrenaria* and *Tutcheria*). Thus, the expanded *Camellia s.l*., which now includes all genera recognized by Keng (1962) as the tribe Camellieae, has been proposed [4]. Indeed, most of the genera referred to above have at times been included within *Camellia*. For example, *Tutcheria* has been established based on *Camellia spectabilis* Champ., a specimen from Hong Kong. Another case is *Glytocarpa* Hu. It once belonged to the genus *Camellia* (*Kailosocarpus camellioides* Hu), but was later recognized as either a member of *Pyrenaria* [67] or a member of *Camellia* [1], [14]. Earlier authors emphasized floral characters, such as the number and the degree of differentiation among bracteoles, sepals and petals [8], [9], to delimit *Camellia*. Subsequent classification systems focused on fruit morphology and seed characters, such as the type, size, shape and the presence or absence of columella [4], [10]. All of these characters, however, have been proved to be plesiomophic [22]. In fact, *Pyrenaria s*.*l*. and *Camellia* are so similar in morphology that it is rather difficult to distinguish one from the other, and therefore misidentifications between these two genera are frequent (e.g. Figure S2).

Many of the outstanding taxonomic problems in *Camellia* have often been attributed to the heavy reliance on plesiomophic and/or homoplastic characters [e.g. 11,22,68]. However, the conflicting results between our cpDNA sequences and nuclear gene sequences indicated that hybridization had once widely occurred between *Camellia* and *Pyrenaria s.l*. The chromosome numbers for *Camellia* and *Pyrenaria* were both found to be 2n = 30, which made it possible for them to cross and the two genera have been successfully crossed in artificial hybridization studies [22], [69], [70]. *Camellia* and *Pyrenaria* have sympatric distributions, currently across southern China and northern Indochina [2], [14], although further fossil evidence may broaden their historical distribution and overlap. Moreover, molecular clock analysis has indicated that they are contemporaries, as both lineages evolved and diversified in the Middle Miocene period (Table 2; Figure 3). Thus hybridization between *Camellia* and *Pyrenaria s.l*. may be one of the potential reasons for their confusing taxonomy.

### Potential hybrid origin of *Apterosperma*

The monotypic *Apterosperma* is endemic to southern China. *Apterosperma oblate* is of public concern and is listed as a protected plant due to the high risk of its extinction in the wild [71]–[75]. The phylogenetic position of *Apterosperma*, however, is unclear and has long been controversial. *Apterosperma* was originally placed in the Gordonieae ( = Schimeae) and considered to be closely related to the genus *Schima* based on the similarities between their embryological and fruit features, such as a higher degree of carpellary congenital fusion, axile-central placentation, the entire stigma and the depressed globose capsules with long columella [11], [12]. Subsequent cytological studies transferred it into tribe Theeae because its chromosome number of 2n  = 30 differed from *Schima* where 2n = 36 [19]. Palynological data, however, showed the pollen of *Apterosperma* to have a smooth wall, which differs from other Theaceae genera, thus suggesting a separate tribe [76]. Previous molecular evidence confirmed its placement within Theeae, but its position was still controversial [15], [16]. Our results suggested that *Apterosperma* was clustered together with clade *Polyspora* (IB) or *Parapyrenaria* (IID), based on the cpDNA data and nuclear data, respectively. The findings suggest that *Apterosperma* may be an ancient hybrid from a maternal parent of *Polyspora* and a paternal parent of *Pyrenaria s.l*. (*Parapyrenaria*). Moreover, *Apterosperma* has two kinds of *LEAFY* sequences that are 1016 bp and 1243 bp in length, respectively. Phylogenetic analyses showed that they are not monophyletic. One copy clustered with *Parapyrenaria*, whereas the other copy was sister to all remaining accessions in the clade. Furthermore, the molecular clock data showed that the two copies are not contemporaneous. One copy occurred at about 2.56 mya (95% HPD: 1.29–5.95 mya), while the other copy, sister to all remaining accessions in the clade, occurred at about 21.10 mya, (95% HPD: 14.91–21.48 mya), which is concurrent with that of *Polyspora* (Figure 3). The topological results among different gene trees indicated that repeated backcrosses have occurred between the ancient hybrids (♀) and the *Parapyrenaria* species (♂) (introgression from the *Parapyrenaria* lineage), thus the *Polyspora LEAFY* copy has been gradually replaced by the copy from *Parapyrenaria* species. These evolutionary processes can explain why there was one copy in the chloroplast data, and why the *Polyspora* copy has not been found in the existing two *LEAFY* copies.

Genetic variation levels were high in *Apterosperma* [74], which is in contrast with the general idea that small populations of endangered species have low genetic diversity [77]. However, taking its potential hybrid origin into consideration, this discrepancy is not surprising. The seeds of *Apterosperma* were originally described as wingless. Subsequent investigations, however, found both sterile and fertile seeds in *Apterosperma oblata*. The fertile seed, which resembles *Polyspora* seed, is flat with narrow apical wings, while the sterile seed is Pyrenaria-like and is basally convex-hemispherical with no wings [19]. The intermediate morphological features and the molecular data support the potential hybrid origin of *Apterosperma*.

### Phylogenetic position of *Polyspora*

*Polyspora* was once placed in *Gordonia* based on their overall morphological similarities, such as the elongated capsule and apically winged seeds [4], [8], [10]. Other authors held different views, however, and suggested that the Asian species should be isolated from North American species as a separate *Polyspora* genus [9], [11], [12]. Previous molecular studies confirmed the monophyly of *Polyspora*, but left its position controversial, placing it as either a sister group of *Camellia* [16], [17] or in a basal position of the Theeae clade [15]. Our results, derived from the cpDNA and nuclear *LEAFY* sequence data, confirming its monophyletic and supporting its placement as the sister group to the remaining members of tribe Theeae.

### Conclusions

The systematics of the Theeae is a long-standing problem, resulting in many taxonomic uncertainties at the generic level. This study presents the first phylogenetic study of Theeae based on the nuclear *LEAFY* gene and four cpDNA regions, and hence provides a framework for a systematic revision of these taxa. The results clearly support the monophyly of *Polyspora* and its basal placement in Theeae. In contrast, *Camellia* is paraphyletic and *Pyrenaria s.l*. is polyphyletic. The New World *Laplacea* seems to be sister to the *Pyrenaria s.l*. lineage. These results suggest that there is a need to revise the current classification of Theeae. The significant incongruence between the nuclear and cpDNA datasets in the placement of *Apterosperma*, as well as some species of *Camellia* and *Tutcheria*, suggests widespread hybridization within the Theeae. We hypothesize that hybridization has played an important role in the evolution of Theeae, which may account for the confusing taxonomy of the tribe.

## Supporting Information

Figure S1 **Fossil-calibrated molecular chronogram of Theeae based on the cpDNA sequences.** Dark gray bars represent 95% confident intervals for nodal ages. Numbers adjacent to the nodes indicate the ages of the nodes of interest (also see Table 2).
(JPG)

Figure S2 **A specimen of Theeae that has been identified as a number of different species by alternative authors.**
(JPG)

Table S1 **List of taxa used in analyses, their sources, voucher information and Genbank accession numbers.**
(DOC)

Table S2 **Sequences of primers used for PCR amplification and sequencing.**
(DOC)
