# Supplementary figures and images for: Molecular Phylogeny of Tribe Theeae (Theaceae s.s.) and Its Implications for Generic Delimitation
Source: PLoS One. 2014 May 21;9(5):e98133. doi: 10.1371/journal.pone.0098133 (PMC4029964; doi:10.1371/journal.pone.0098133)

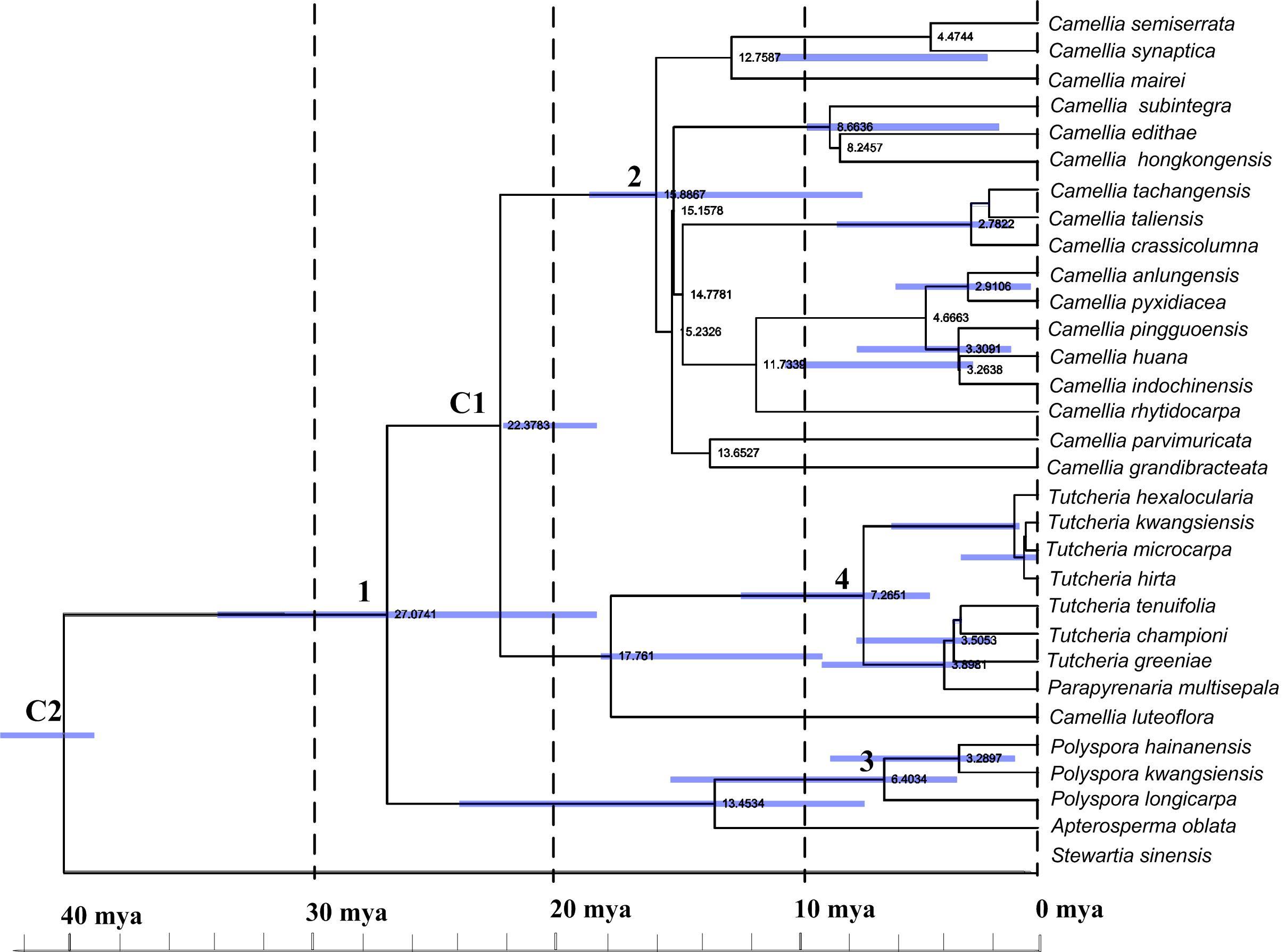

Supplement: Figure S1 — Fossil-calibrated molecular chronogram of Theeae based on the cpDNA sequences. Dark gray bars represent 95% confident intervals for nodal ages. Numbers adjacent to the nodes indicate the ages of the nodes of interest (also see Table 2). (JPG) [file pone.0098133.s001.jpg]

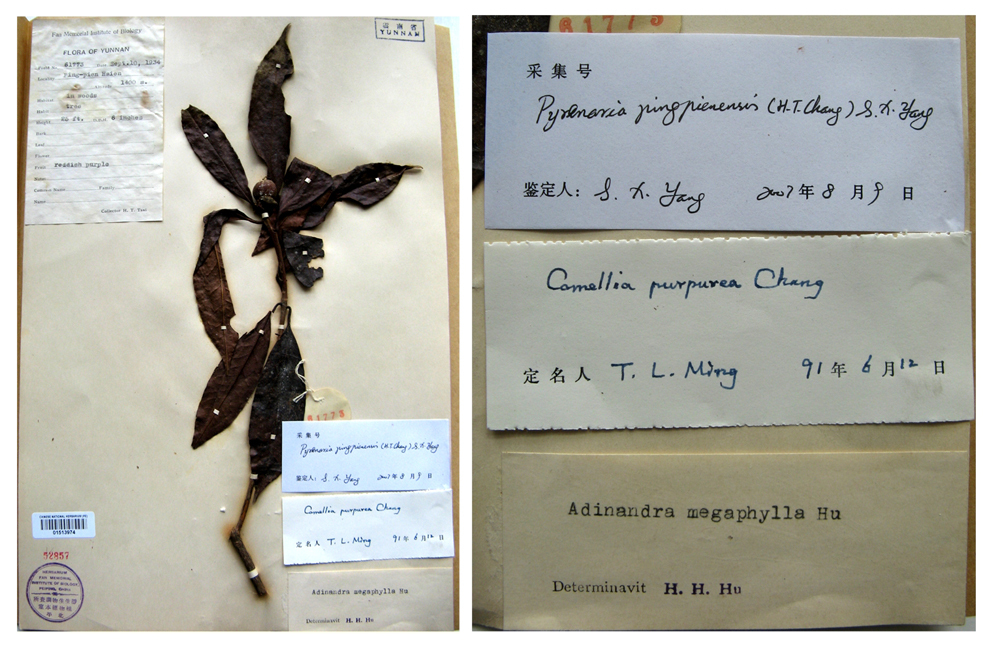

Supplement: Figure S2 — A specimen of Theeae that has been identified as a number of different species by alternative authors. (JPG) [file pone.0098133.s002.jpg]
