# Supplementary material for: Molecular Phylogeny of Tribe Theeae (Theaceae s.s.) and Its Implications for Generic Delimitation
Source: PLoS One. 2014 May 21;9(5):e98133. doi: 10.1371/journal.pone.0098133 (PMC4029964; doi:10.1371/journal.pone.0098133)
Supplement: Table S1 — List of taxa used in analyses, their sources, voucher information and Genbank accession numbers. (DOC) [file pone.0098133.s003.doc]

**Table S1. List of taxa used in analyses, their sources, voucher information and Genbank accession numbers.**

| Taxa | Source | Voucher (herbarum) | GenBank Accession No. | | | | |
| --- | --- | --- | --- | --- | --- | --- | --- |
|  |  |  | *atpH-I* | *matK* | *psbA5’R-ALS-11F* | *rbcL* | *LEAFY* |
| *Apterosperma oblate* Chang | Guangzhou, China | MG Li 09001 (PE) | KJ198033 | KJ197940 | KJ198002 | KJ197971 | KJ197915;  KJ197916;  KJ197917;  KJ197918 |
| *Camellia albigogas* Hu | Genbank |  |  | AF380072 |  | AF380033 |  |
| *Camellia anlungensis* Chang | Jinhua, Zhejiang, China | W Zhang 806036 (PE) | KJ198013 | KJ197920 | KJ197982 | KJ197951 | KJ197881 |
| *Camellia crassicolumna* Chang | Xichou, Yunnan, China | H Jang 03904 (PE) | KJ198019 | KJ197926 | KJ197988 | KJ197957 | KJ197887;  KJ197888 |
| *Camellia edithae* Hance | Jinhua, Zhejiang, China | W Zhang 806180 (PE) | KJ198025 | KJ197932 | KJ197994 | KJ197963 | KJ197885;  KJ197886 |
| *Camellia* *grandibracteata* Chang & Yu | Menghai, Yunnan, China | Mei Xu060626-2 (PE) | KJ198029 | KJ197936 | KJ197998 | KJ197967 | KJ197875; KJ197876 |
| *Camellia granthamiana* Sealy | Genbank |  |  | AF380073 |  | AF380034 |  |
| *Camellia* *hongkongensis* Seem. | Jinhua, Zhejiang, China | W Zhang 806169 (PE) | KJ198024 | KJ197931 | KJ197993 | KJ197962 | KJ197884 |
| *Camellia huana* Ming & Zhang | Jinhua, Zhejiang, China | W Zhang 806097 (PE) | KJ198015 | KJ197922 | KJ197984 | KJ197953 | KJ197895 |
| *Camellia indochinensis* Merrill | Jinhua, Zhejiang, China | W Zhang 806074 (PE) | KJ198023 | KJ197930 | KJ197992 | KJ197961 | KJ197892 |
| *Camellia japonica* L. | Genbank |  |  | AF380074 |  | AF380035 |  |
| *Camellia luteoflora* Li & Chang | Jinhua, Zhejiang, China | W Zhang 806280 (PE) | KJ198020 | KJ197927 | KJ197989 | KJ197958 | KJ197896;  KJ197897 |
| *Camellia mairei* (Levl.) Melchior | Jinhua, Zhejiang, China | W Zhang 806146 (PE) | KJ198026 | KJ197933 | KJ197995 | KJ197964 | KJ197890 |
| *Camellia parvimuricata* Chang | Jinhua, Zhejiang, China | W Zhang 806029 (PE) | KJ198012 | KJ197919 | KJ197981 | KJ197950 | KJ197879;  KJ197880 |
| *Camellia pingguoensis* D. Fang | Jinhua, Zhejiang, China | W Zhang 806101 (PE) | KJ198016 | KJ197923 | KJ197985 | KJ197954 | KJ197893;  KJ197894 |
| *Camellia pyxidiacea* Xu, Chen & Deng | Xichou, Yunnan, China | ZY Li Y-1-1 (PE) | KJ198022 | KJ197929 | KJ197991 | KJ197960 | KJ197874 |
| *Camellia rhytidocarpa* Chang & Liang | Jinhua, Zhejiang, China | W Zhang 806032 (PE) | KJ198021 | KJ197928 | KJ197990 | KJ197959 | KJ197872; KJ197873 |
| *Camellia sasanqua* Thunberg | Genbank |  |  | AF380076 |  | AF380036 |  |
| *Camellia semiserrata* C.W. Chi | Jinhua, Zhejiang, China | W Zhang 806140 (PE) | KJ198018 | KJ197925 | KJ197987 | KJ197956 | KJ197891 |
| *Camellia sinensis* (L.) O. Kuntze | Genbank |  |  | AF380077 |  | AF380037 |  |
| *Camellia subintegra* Huang & Chang | Jinhua, Zhejiang, China | W Zhang 806158 (PE) | KJ198017 | KJ197924 | KJ197986 | KJ197955 | KJ197883 |
| *Camellia synaptica* Sealy | Jinhua, Zhejiang, China | W Zhang 806052 (PE) | KJ198014 | KJ197921 | KJ197983 | KJ197952 | KJ197882 |
| *Camellia tachangensis* F.C. Zhang | Puan, Guizhhou, China | ZH Deng 4944 (PE) | KJ198028 | KJ197935 | KJ197997 | KJ197966 | KJ197877;  KJ197878 |
| *Camellia taliensis* (Smith) Melch. | Xichou, Yunnan, China | H Jiang 3885 (PE) | KJ198027 | KJ197934 | KJ197996 | KJ197965 | KJ197889; |
| *Laplacea fruticosa* Kobuski | Genbank |  |  | AF380088 |  | AF380045 |  |
| *Laplacea portoricensis* Dyer | Genbank |  |  | AF380089 |  | AF380046 |  |
| *Parapyrenaria multisepala* (Merr.& Chun) Chang | Wanning, Hainan, China | GT Li 09001 (PE) | KJ198042 | KJ197949 | KJ198011 | KJ197980 | KJ197913;  KJ197914 |
| *Polyspora axillaris* (Roxb.) Sweet | Genbank |  |  | AF380090 |  | AF380047 |  |
| *Polyspora chrysandra* (Cowan) Ye | Genbank |  |  | AF380091 |  | AF380048 |  |
| *Polyspora hainanensis* Chang | Changsha, Hunan, China | W Zhang 806284 (PE) | KJ198031 | KJ197938 | KJ198000 | KJ197969 | KJ197907;  KJ197908 |
| *Polyspora hainanensis* Chang | Genbank |  |  | AF380093 |  | AF380050 |  |
| *Polyspora kwangsiensis* Chang | Changsha, Hunan China | W Zhang 806291 (PE) | KJ198032 | KJ197939 | KJ198001 | KJ197970 | KJ197905 |
| *Polyspora longicarpa* Chang | Kunming, Yunnan, China | X Yang090501 | KJ198030 | KJ197937 | KJ197999 | KJ197968 | KJ197906 |
| *Polyspora yunnanensis* Hu | Genbank |  |  | AF380095 |  | AF380052 |  |
| *Stewartia sinensis* Rehder & Wilson | Kunming, Yunnan, China | X Yang090506 | KJ198036 | KJ197943 | KJ198005 | KJ197974 | KJ197909;  KJ197910 |
| *Tutcheria championi* Nakai | Shangsi, Guangxi, China | ZL Ning 10002 (PE) | KJ198035 | KJ197942 | KJ198004 | KJ197973 | KJ197911  KJ197912 |
| *Tutcheria greeniae* Chun | Shangsi, Guangxi, China | ZL Ning 10320 (PE) | KJ198037 | KJ197944 | KJ198006 | KJ197975 | KJ197901 |
| *Tutcheria hexalocularia* Hu & Liang ex Chang | Shangsi, Guangxi, China | ZL Ning 10003 (PE) | KJ198039 | KJ197946 | KJ198008 | KJ197977 | KJ197902 |
| *Tutcheria hirta* (Hand.-Mazz.) Li | Shangsi, Guangxi, China | ZL Ning 10321 (PE) | KJ198034 | KJ197941 | KJ198003 | KJ197972 | KJ197904 |
| *Tutcheria kwangsiensis* (Chang) Chang & Ye | Shangsi, Guangxi, China | ZL Ning 10004 (PE) | KJ198040 | KJ197947 | KJ198009 | KJ197978 | KJ197903 |
| *Tutcheria microcarpa* Dunn | Changsha, Hunan, China | W Zhang 09300 (PE) | KJ198038 | KJ197945 | KJ198007 | KJ197976 | KJ197898;  KJ197899 |
| *Tutcheria tenuifolia* Chang | Shangsi, Guangxi, China | ZL Ning 10005 (PE) | KJ198041 | KJ197948 | KJ198010 | KJ197979 | KJ197900 |
