# Supplementary material for: Molecular Phylogeny of Tribe Theeae (Theaceae s.s.) and Its Implications for Generic Delimitation
Source: PLoS One. 2014 May 21;9(5):e98133. doi: 10.1371/journal.pone.0098133 (PMC4029964; doi:10.1371/journal.pone.0098133)
Supplement: Table S2 — Sequences of primers used for PCR amplification and sequencing. (DOC) [file pone.0098133.s004.doc]

**Table S2. Sequences of primers used for PCR amplification and sequencing**

| Region | Name | Primer sequence (5’-3-) | Source |
| --- | --- | --- | --- |
| *atpI-atpH* | atpI | TATTTACAAGYGGTATTCAAGCT | [34] |
|  | atpH | CCAAYCCAGCAGCAATAAC |  |
| *ALS*-*11F*-*psbA5’R* | psbA5’R | AACCATCCAATGTAAAGACGGTTT | [33] |
|  | ALS-11F | ATCTTTCGCATTATTATAG |  |
| *matK* | matK-F | TCAGGAGTATATTTATGCACTTGCT | This study |
|  | matK-R2 | CTCAGTTGATTTAAGCCTTACTACAT |  |
| *rbcL* | rbcL-F | TGGACCGATGGACTTATCAGCCTTG | This study |
|  | rbcL-R | TCCTTCCATACCTCACAAGCAGCAG |  |
| *LEAFY* | LFY-F | TACCTCTTCCATCTCTACGAGCAGT | This study |
|  | LFY-R | ACCGGCCTTCTTGGCGTACCT |  |
